# Supplementary material for: A Bayesian adaptive design for dual‐agent phase I–II oncology trials integrating efficacy data across stages
Source: Biom J. 2023 May 18;65(7):2200288. doi: 10.1002/bimj.202200288 (PMC10952513; doi:10.1002/bimj.202200288)

A Bayesian adaptive design for dual-agent phase I-II oncology trials integrating efficacy data across stages: supporting information

José L. Jiménez[1*] and Haiyan Zheng[2]
[1]Novartis Pharma A.G., Basel, Switzerland
[2]MRC Biostatistics Unit, University of Cambridge, Cambridge, U.K.
[*]jose_luis.jimenez@novartis.com

Table S1: Median prior probability of efficacy with 95% credible intervals using weakly informative prior distributions

Cisplatin (mg/m^2)	Cabazitaxel (mg/m^2)	Median (95% Credible Interval)		
		$w = 0$	$w = 0.5$	$w = 1$
10	50	0.14 (0 - 0.99)	0.14 (0 - 0.99)	0.14 (0 - 0.99)
10	100	0.41 (0 - 1)	0.41 (0 - 1)	0.42 (0 - 1)
25	50	0.42 (0 - 1)	0.42 (0 - 1)	0.42 (0 - 1)
25	100	0.84 (0 - 1)	0.85 (0 - 1)	0.86 (0 - 1)
17.5	75	0.48 (0 - 1)	0.5 (0 - 1)	0.5 (0 - 1)
15	75	0.41 (0 - 1)	0.41 (0 - 1)	0.41 (0 - 1)

Table S2: True dose-toxicity model parameter values of the 2 scenarios taken from Tighiouart (2019).

Stage I & II dose-toxicity profile parameters		
	Profile 1	Profile 2
ρ_{00}	1×10^{-7}	0.001
ρ_{01}	0.2	0.05
ρ_{10}	0.2	0.05
α_3	10	10

We recall that, to facilitate the comprehension of the simulation study, we rename the dose-toxicity and dose-efficacy scenarios as follows:

- Dose-toxicity profile 1 + stage II dose-efficacy profile 1 under H_1 = Scenario A,
- Dose-toxicity profile 1 + stage II dose-efficacy profile 2 under H_1 = Scenario B,
- Dose-toxicity profile 2 + stage II dose-efficacy profile 1 under H_1 = Scenario C,
- Dose-toxicity profile 2 + stage II dose-efficacy profile 2 under H_1 = Scenario D,
- Dose-toxicity profile 1 + stage II dose-efficacy profile 1 under H_0 = Scenario E,
- Dose-toxicity profile 1 + stage II dose-efficacy profile 2 under H_0 = Scenario F,
- Dose-toxicity profile 2 + stage II dose-efficacy profile 1 under H_0 = Scenario G,
- Dose-toxicity profile 2 + stage II dose-efficacy profile 2 under H_0 = Scenario H.

Table S3: True dose-efficacy model parameter values for stage I and II scenarios under H_1.

	Stage I dose-efficacy paramters					Stage II dose-efficacy parameters			
	Agreement level	β_{01}	β_{11}	β_{21}	β_{23}	β_{02}	β_{12}	β_{22}	β_{32}
Scenario A	CA	-5	0.75	1.51	0.5	-5	0.75	1.51	0.5
	PA	-5	0.35	1.11	0.5				
	CD	-5	1.31	0.75	0.5				
Scenario B	CA	-5	1.5035	1.1	0.5	-5	1.5035	1.1	0.5
	PA	-5	1.5	0.2	0.5				
	CD	-8	-10	-10	0				
Scenario C	CA	-6	1.2	1.623	0	-6	1.2	1.623	0
	PA	-6	1.4	1.6	0				
	CD	-6	1.623	1.2	0				
Scenario D	CA	-4	1.025	0.7	3	-4	1.025	0.7	3
	PA	-4.5	1.025	0.7	3				
	CD	-8	-5	-5	27				

Table S4: True dose-efficacy model parameter values for stage I and II scenarios under H_0.

	Stage I dose-efficacy paramters					Stage II dose-efficacy parameters			
	Agreement level	β_{01}	β_{11}	β_{21}	β_{23}	β_{02}	β_{12}	β_{22}	β_{32}
Scenario E	CA	-4	-2	0.8	0.5	-4	-2	0.8	0.5
	PA	-4	-2	0.1	1				
	CD	-4.5	1.4	1	0.5				
Scenario F	CA	-6.36	1.5035	1.1	0.5	-6.36	1.5035	1.1	0.5
	PA	-6.36	1.65	1.1	0.5				
	CD	-1.5	-1	-1	0.25				
Scenario G	CA	-6	1.1	1.323	0	-6	1.1	1.323	0
	PA	-7	1.1	1.4	0				
	CD	-6	1.622	1.2	0				
Scenario H	CA	-5.35	1.025	0.7	3	-5.35	1.025	0.7	3
	PA	-5	1.1	-1	1				
	CD	-3.54	0.5	1	1				

Table S5: Average sample size under the early stopping for futility rule under H_1 (Scenarios A-D) and H_0 (Scenarios E-H).

	Scenario A			Scenario B			Scenario C			Scenario D		
	CA	PA	CD	CA	PA	CD	CA	PA	CD	CA	PA	CD
$\omega = 0$	53	53	53	55	55	55	53	53	53	55	55	55
$\omega = 0.25$	53	53	54	55	55	55	53	53	53	55	55	55
$\omega = 0.5$	54	54	54	55	55	55	54	54	54	55	55	55
$\omega = 0.75$	54	54	54	55	55	55	54	54	54	55	55	55
$\omega = 1$	54	54	54	55	55	55	54	54	54	55	55	55
	Scenario E			Scenario F			Scenario G			Scenario H		
	CA	PA	CD	CA	PA	CD	CA	PA	CD	CA	PA	CD
$\omega = 0$	45	45	45	46	46	46	46	46	46	47	47	47
$\omega = 0.25$	46	46	46	47	47	47	47	47	47	48	48	49
$\omega = 0.5$	46	46	47	47	47	47	47	47	48	48	48	48
$\omega = 0.75$	46	46	47	48	48	47	48	47	48	49	49	49
$\omega = 1$	47	47	47	48	48	47	48	48	48	49	49	49

Figure S1: Probability of rejecting H_0 in scenarios under H_1 (i.e., power) and H_0 (i.e., type-I error). Scenario A-D and E-H are scenario under H_1 and H_0, respectively.

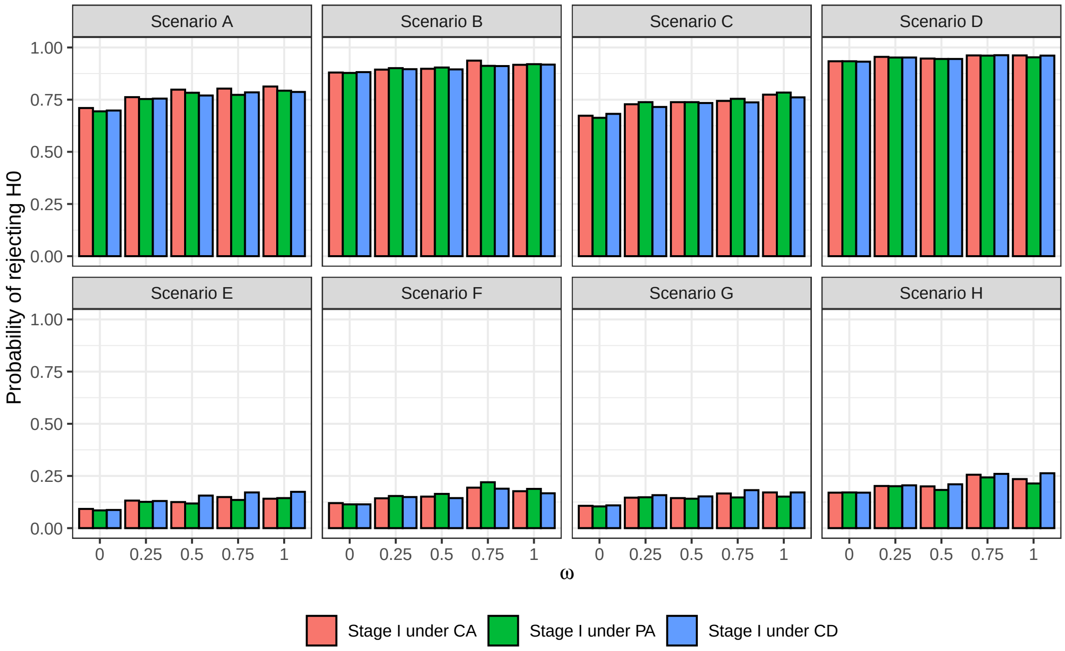

Figure S2: Difference in the proportion of patients allocated to dose combination with true probability of efficacy above p_0 (i.e., patients correctly allocated) in stage II with $\omega > 0$ with respect to $\omega = 0$.

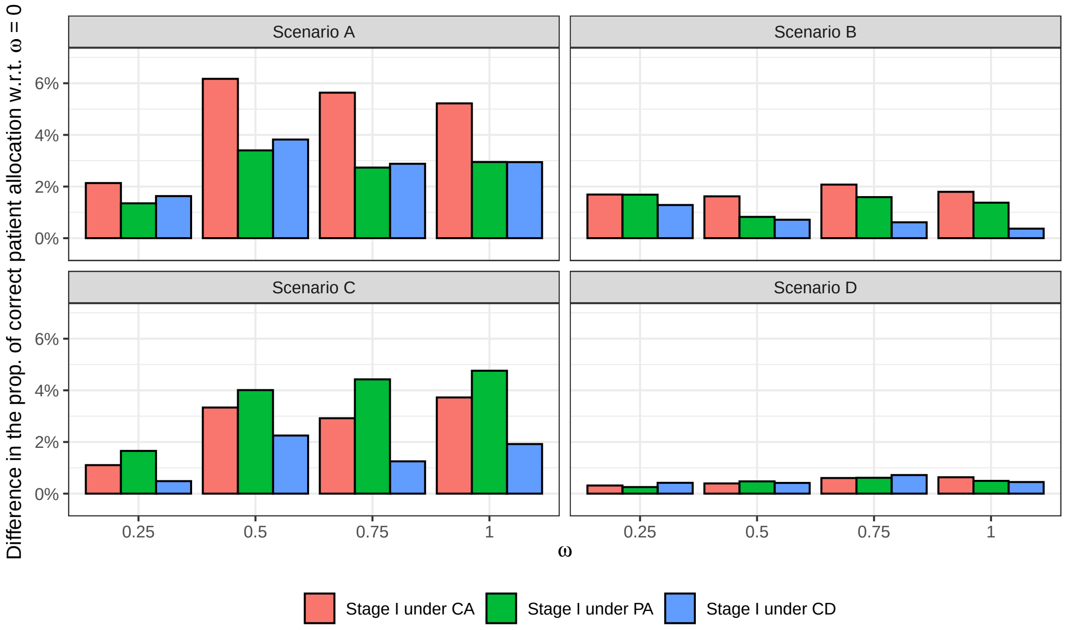

Figure S3: Probability of early stopping for safety.

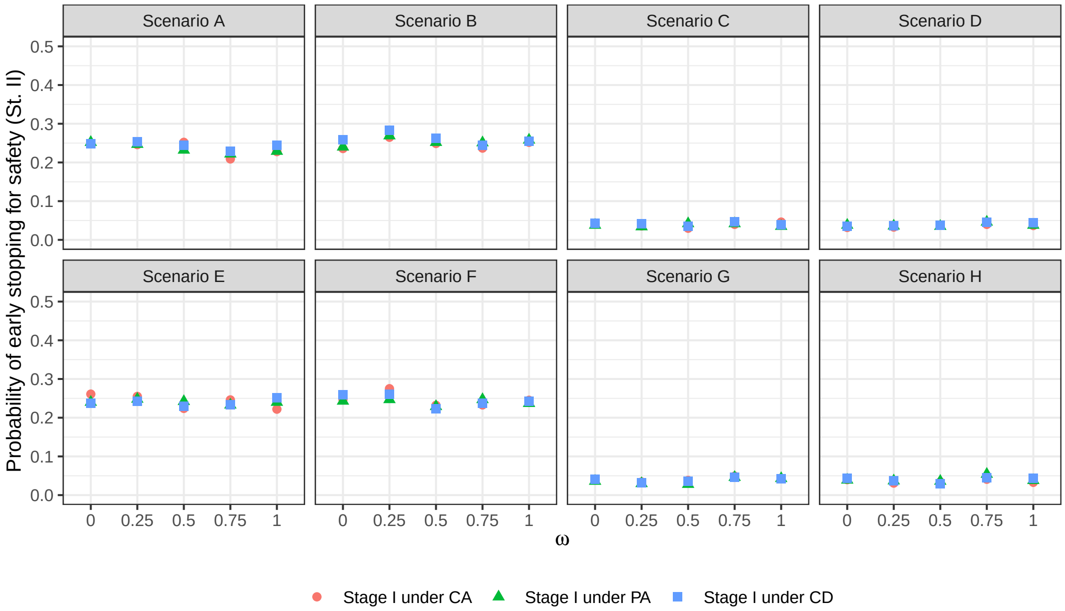

Supplement: Supplementary file 2 — Supporting Information S2 [file BIMJ-65-0-s002.pdf]
