# Supplementary material for: A Bayesian adaptive design for dual‐agent phase I–II oncology trials integrating efficacy data across stages
Source: Biom J. 2023 May 18;65(7):2200288. doi: 10.1002/bimj.202200288 (PMC10952513; doi:10.1002/bimj.202200288)
Supplement: Supplementary file 1 — Supporting Information S1 [file BIMJ-65-0-s001.zip › Code_biometrical_journal2/Code_biometrical_journal/Results/Figure_1.pdf]

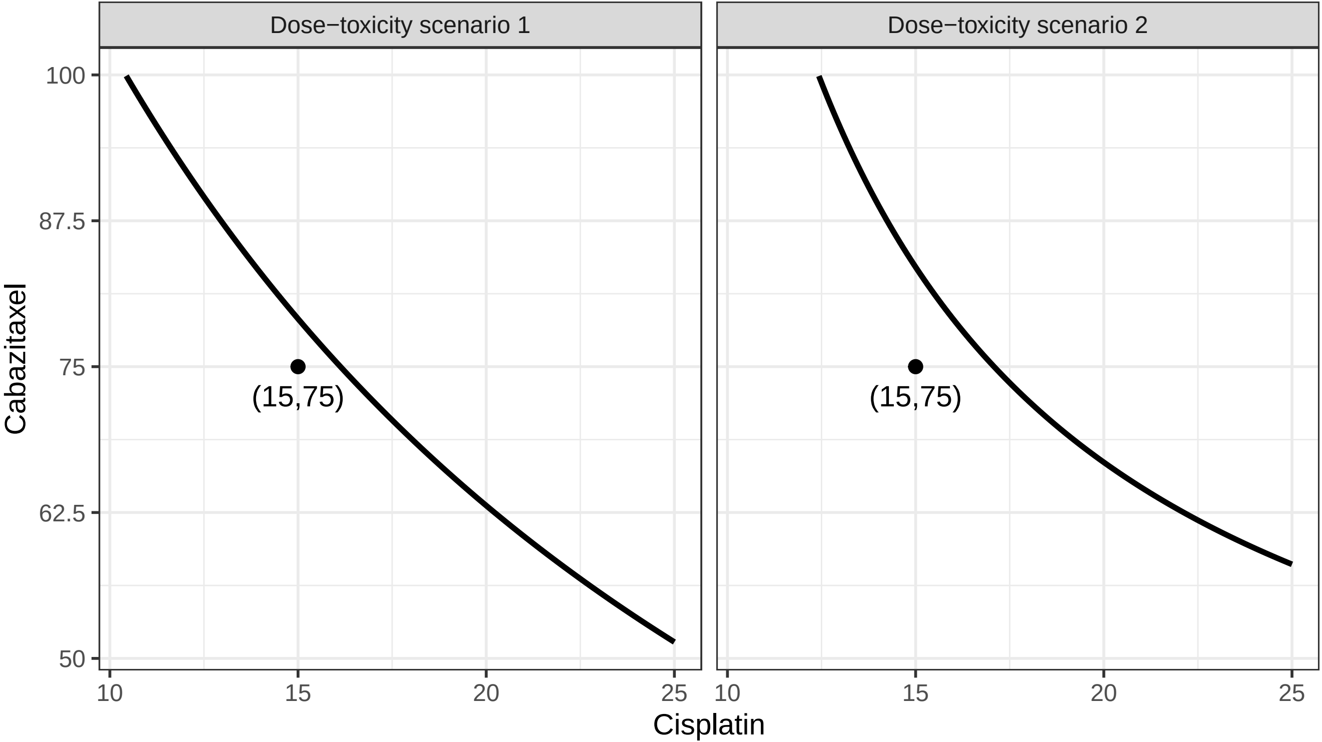
Dose−toxicity scenario 1
Dose−toxicity scenario 2
100
87.5
75
62.5
50
(15,75)
(15,75)
10
15
20
25
10
15
20
25
Cabazitaxel
Cisplatin
