# Supplementary material for: A Bayesian adaptive design for dual‐agent phase I–II oncology trials integrating efficacy data across stages
Source: Biom J. 2023 May 18;65(7):2200288. doi: 10.1002/bimj.202200288 (PMC10952513; doi:10.1002/bimj.202200288)
Supplement: Supplementary file 1 — Supporting Information S1 [file BIMJ-65-0-s001.zip › Code_biometrical_journal2/Code_biometrical_journal/Results/Figure_4.pdf]

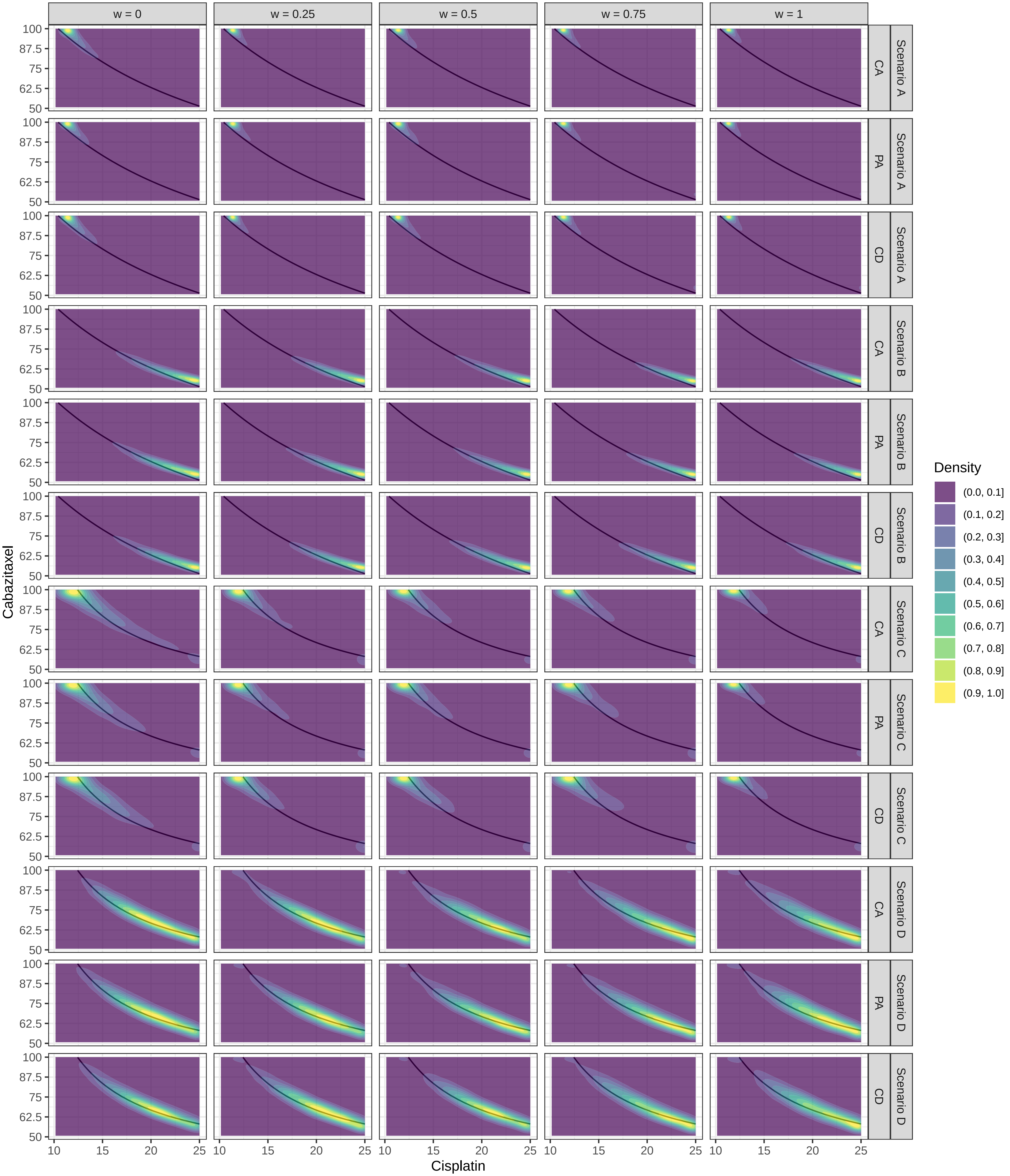

w = 0
w = 0.25
w = 0.5
w = 0.75
w = 1
Scenario A
Scenario B
Scenario C
Scenario D
CA
PA
CD
Cabazitaxel
Cisplatin
100
87.5
75
62.5
50
10
15
20
25
Density
(0.0, 0.1]
(0.1, 0.2]
(0.2, 0.3]
(0.3, 0.4]
(0.4, 0.5]
(0.5, 0.6]
(0.6, 0.7]
(0.7, 0.8]
(0.8, 0.9]
(0.9, 1.0]
