# Supplementary material for: A Bayesian adaptive design for dual‐agent phase I–II oncology trials integrating efficacy data across stages
Source: Biom J. 2023 May 18;65(7):2200288. doi: 10.1002/bimj.202200288 (PMC10952513; doi:10.1002/bimj.202200288)
Supplement: Supplementary file 1 — Supporting Information S1 [file BIMJ-65-0-s001.zip › Code_biometrical_journal2/Code_biometrical_journal/Results/Figure_6.pdf]

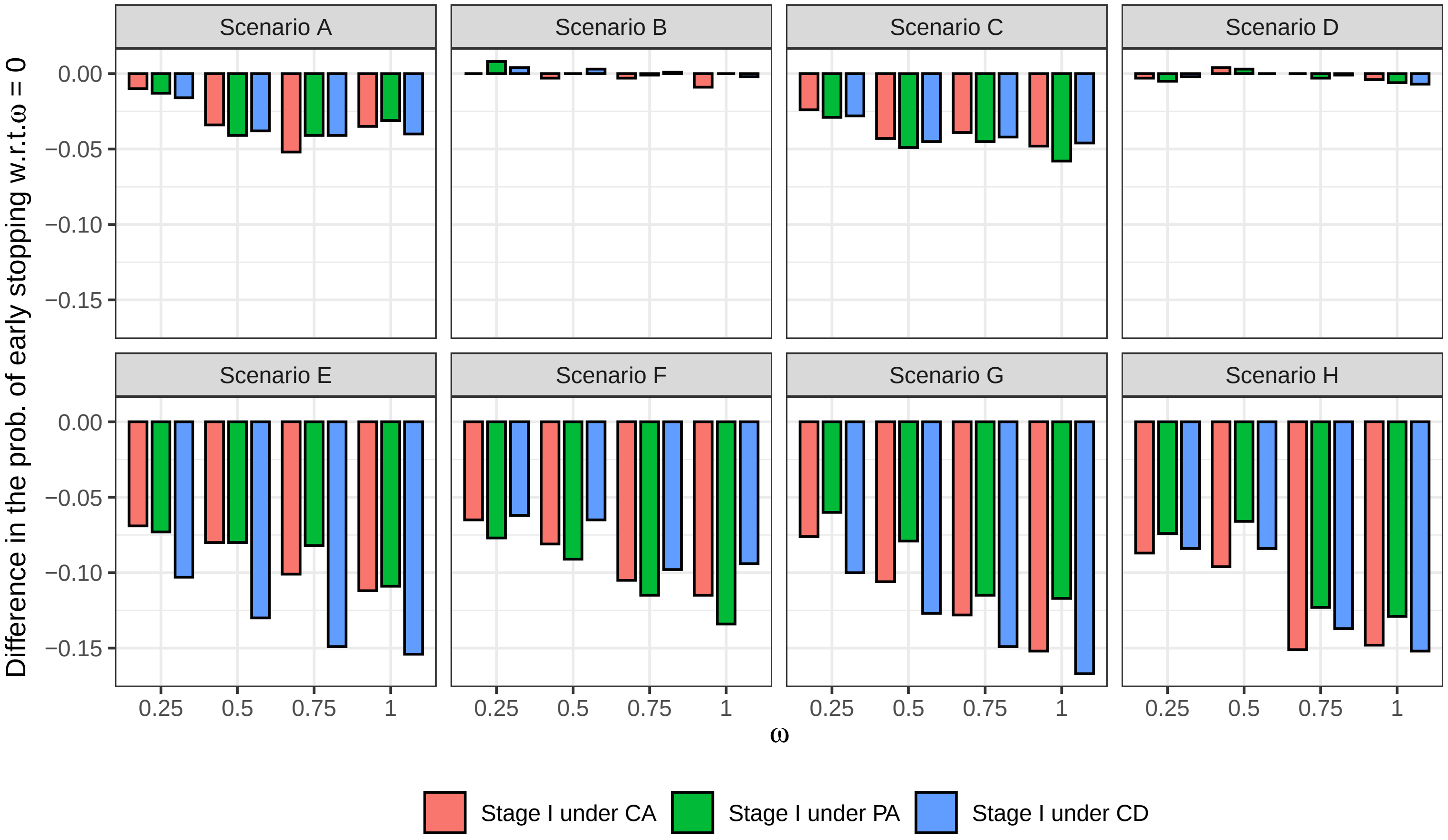
Scenario A
Scenario B
Scenario C
Scenario D
Scenario E
Scenario F
Scenario G
Scenario H
Difference in the prob. of early stopping w.r.t.ω = 0
0.00
−0.05
−0.10
−0.15
0.25
0.5
0.75
1
ω
Stage I under CA
Stage I under PA
Stage I under CD
