# Supplementary material for: A Bayesian adaptive design for dual‐agent phase I–II oncology trials integrating efficacy data across stages
Source: Biom J. 2023 May 18;65(7):2200288. doi: 10.1002/bimj.202200288 (PMC10952513; doi:10.1002/bimj.202200288)
Supplement: Supplementary file 1 — Supporting Information S1 [file BIMJ-65-0-s001.zip › Code_biometrical_journal2/Code_biometrical_journal/Results/Figure_S1.pdf]

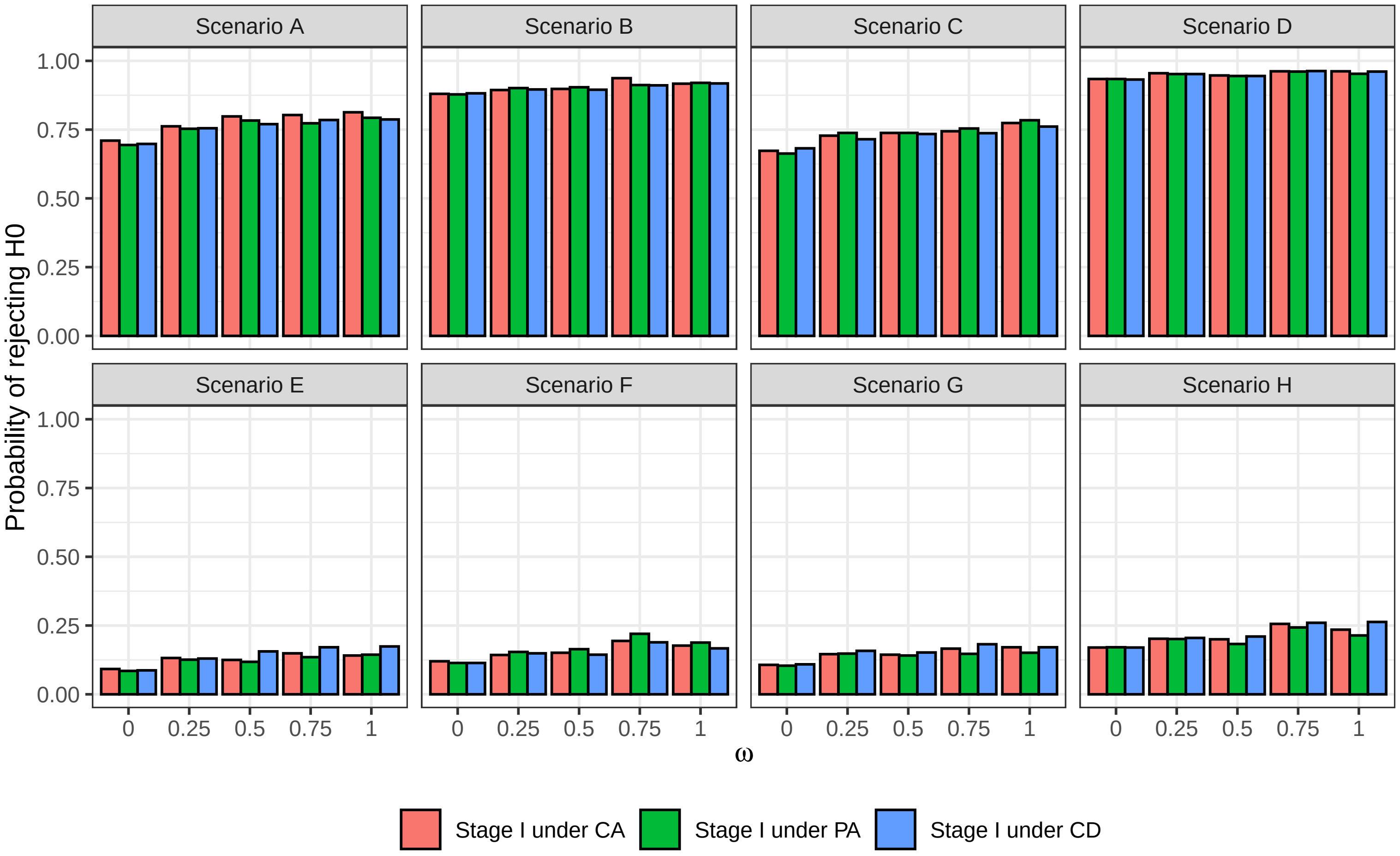

Scenario A
Scenario B
Scenario C
Scenario D
Scenario E
Scenario F
Scenario G
Scenario H
Probability of rejecting H0
1.00
0.75
0.50
0.25
0.00
0
0.25
0.5
0.75
1
ω
Stage I under CA
Stage I under PA
Stage I under CD
