# Supplementary material for: A Bayesian adaptive design for dual‐agent phase I–II oncology trials integrating efficacy data across stages
Source: Biom J. 2023 May 18;65(7):2200288. doi: 10.1002/bimj.202200288 (PMC10952513; doi:10.1002/bimj.202200288)
Supplement: Supplementary file 1 — Supporting Information S1 [file BIMJ-65-0-s001.zip › Code_biometrical_journal2/Code_biometrical_journal/Results/Table_S1.pdf]

	x	y	omega	median	CI95_lower	CI95_upper
1	10	50	0	0.14	0	0.99
2	10	75	0	0.26	0	1
3	10	100	0	0.42	0	1
4	15	50	0	0.22	0	0.99
5	15	75	0	0.41	0	1
6	15	100	0	0.61	0	1
7	17.5	50	0	0.26	0	1
8	17.5	75	0	0.49	0	1
9	17.5	100	0	0.68	0	1
10	25	50	0	0.41	0	1
11	25	75	0	0.68	0	1
12	25	100	0	0.84	0	1
13	10	50	0.5	0.14	0	0.99
14	10	75	0.5	0.27	0	1
15	10	100	0.5	0.43	0	1
16	15	50	0.5	0.22	0	0.99
17	15	75	0.5	0.43	0	1
18	15	100	0.5	0.63	0	1
19	17.5	50	0.5	0.27	0	1
20	17.5	75	0.5	0.51	0	1
21	17.5	100	0.5	0.71	0	1
22	25	50	0.5	0.43	0	1
23	25	75	0.5	0.71	0	1
24	25	100	0.5	0.86	0	1
25	10	50	1	0.14	0	0.99
26	10	75	1	0.28	0	1
27	10	100	1	0.44	0	1
28	15	50	1	0.23	0	1
29	15	75	1	0.45	0	1
30	15	100	1	0.65	0	1
31	17.5	50	1	0.28	0	1
32	17.5	75	1	0.53	0	1
33	17.5	100	1	0.73	0	1
34	25	50	1	0.43	0	1
35	25	75	1	0.73	0	1
36	25	100	1	0.88	0	1
