# Supplementary material for: A Bayesian adaptive design for dual‐agent phase I–II oncology trials integrating efficacy data across stages
Source: Biom J. 2023 May 18;65(7):2200288. doi: 10.1002/bimj.202200288 (PMC10952513; doi:10.1002/bimj.202200288)
Supplement: Supplementary file 1 — Supporting Information S1 [file BIMJ-65-0-s001.zip › Code_biometrical_journal2/Code_biometrical_journal/Results/Table_S5.pdf]

	DLT_scenario	EFF_scenario	omega	agreement	hypothesis	sample_size
1	Dose−toxicity scenario 1	Dose−efficacy scenario 1	0.00	CA	H1	53
2	Dose−toxicity scenario 1	Dose−efficacy scenario 1	0.25	CA	H1	53
3	Dose−toxicity scenario 1	Dose−efficacy scenario 1	0.50	CA	H1	54
4	Dose−toxicity scenario 1	Dose−efficacy scenario 1	0.75	CA	H1	54
5	Dose−toxicity scenario 1	Dose−efficacy scenario 1	1.00	CA	H1	54
6	Dose−toxicity scenario 1	Dose−efficacy scenario 1	0.00	PA	H1	53
7	Dose−toxicity scenario 1	Dose−efficacy scenario 1	0.25	PA	H1	53
8	Dose−toxicity scenario 1	Dose−efficacy scenario 1	0.50	PA	H1	54
9	Dose−toxicity scenario 1	Dose−efficacy scenario 1	0.75	PA	H1	54
10	Dose−toxicity scenario 1	Dose−efficacy scenario 1	1.00	PA	H1	54
11	Dose−toxicity scenario 1	Dose−efficacy scenario 1	0.00	CD	H1	53
12	Dose−toxicity scenario 1	Dose−efficacy scenario 1	0.25	CD	H1	54
13	Dose−toxicity scenario 1	Dose−efficacy scenario 1	0.50	CD	H1	54
14	Dose−toxicity scenario 1	Dose−efficacy scenario 1	0.75	CD	H1	54
15	Dose−toxicity scenario 1	Dose−efficacy scenario 1	1.00	CD	H1	54
16	Dose−toxicity scenario 1	Dose−efficacy scenario 2	0.00	CA	H1	55
17	Dose−toxicity scenario 1	Dose−efficacy scenario 2	0.25	CA	H1	55
18	Dose−toxicity scenario 1	Dose−efficacy scenario 2	0.50	CA	H1	55
19	Dose−toxicity scenario 1	Dose−efficacy scenario 2	0.75	CA	H1	55
20	Dose−toxicity scenario 1	Dose−efficacy scenario 2	1.00	CA	H1	55
21	Dose−toxicity scenario 1	Dose−efficacy scenario 2	0.00	PA	H1	55
22	Dose−toxicity scenario 1	Dose−efficacy scenario 2	0.25	PA	H1	55
23	Dose−toxicity scenario 1	Dose−efficacy scenario 2	0.50	PA	H1	55
24	Dose−toxicity scenario 1	Dose−efficacy scenario 2	0.75	PA	H1	55
25	Dose−toxicity scenario 1	Dose−efficacy scenario 2	1.00	PA	H1	55
26	Dose−toxicity scenario 1	Dose−efficacy scenario 2	0.00	CD	H1	55
27	Dose−toxicity scenario 1	Dose−efficacy scenario 2	0.25	CD	H1	55
28	Dose−toxicity scenario 1	Dose−efficacy scenario 2	0.50	CD	H1	55
29	Dose−toxicity scenario 1	Dose−efficacy scenario 2	0.75	CD	H1	55
30	Dose−toxicity scenario 1	Dose−efficacy scenario 2	1.00	CD	H1	55
31	Dose−toxicity scenario 2	Dose−efficacy scenario 1	0.00	CA	H1	53
32	Dose−toxicity scenario 2	Dose−efficacy scenario 1	0.25	CA	H1	53
33	Dose−toxicity scenario 2	Dose−efficacy scenario 1	0.50	CA	H1	54
34	Dose−toxicity scenario 2	Dose−efficacy scenario 1	0.75	CA	H1	54
35	Dose−toxicity scenario 2	Dose−efficacy scenario 1	1.00	CA	H1	54
36	Dose−toxicity scenario 2	Dose−efficacy scenario 1	0.00	PA	H1	53
37	Dose−toxicity scenario 2	Dose−efficacy scenario 1	0.25	PA	H1	53
38	Dose−toxicity scenario 2	Dose−efficacy scenario 1	0.50	PA	H1	54
39	Dose−toxicity scenario 2	Dose−efficacy scenario 1	0.75	PA	H1	54
40	Dose−toxicity scenario 2	Dose−efficacy scenario 1	1.00	PA	H1	54
41	Dose−toxicity scenario 2	Dose−efficacy scenario 1	0.00	CD	H1	53
42	Dose−toxicity scenario 2	Dose−efficacy scenario 1	0.25	CD	H1	53
43	Dose−toxicity scenario 2	Dose−efficacy scenario 1	0.50	CD	H1	54
44	Dose−toxicity scenario 2	Dose−efficacy scenario 1	0.75	CD	H1	54
45	Dose−toxicity scenario 2	Dose−efficacy scenario 1	1.00	CD	H1	54
46	Dose−toxicity scenario 2	Dose−efficacy scenario 2	0.00	CA	H1	55
47	Dose−toxicity scenario 2	Dose−efficacy scenario 2	0.25	CA	H1	55
48	Dose−toxicity scenario 2	Dose−efficacy scenario 2	0.50	CA	H1	55
49	Dose−toxicity scenario 2	Dose−efficacy scenario 2	0.75	CA	H1	55
50	Dose−toxicity scenario 2	Dose−efficacy scenario 2	1.00	CA	H1	55
51	Dose−toxicity scenario 2	Dose−efficacy scenario 2	0.00	PA	H1	55
52	Dose−toxicity scenario 2	Dose−efficacy scenario 2	0.25	PA	H1	55
53	Dose−toxicity scenario 2	Dose−efficacy scenario 2	0.50	PA	H1	55
54	Dose−toxicity scenario 2	Dose−efficacy scenario 2	0.75	PA	H1	55
55	Dose−toxicity scenario 2	Dose−efficacy scenario 2	1.00	PA	H1	55
56	Dose−toxicity scenario 2	Dose−efficacy scenario 2	0.00	CD	H1	55
57	Dose−toxicity scenario 2	Dose−efficacy scenario 2	0.25	CD	H1	55
58	Dose−toxicity scenario 2	Dose−efficacy scenario 2	0.50	CD	H1	55
59	Dose−toxicity scenario 2	Dose−efficacy scenario 2	0.75	CD	H1	55
60	Dose−toxicity scenario 2	Dose−efficacy scenario 2	1.00	CD	H1	55
61	Dose−toxicity scenario 1	Dose−efficacy scenario 1	0.00	CA	H0	45
62	Dose−toxicity scenario 1	Dose−efficacy scenario 1	0.25	CA	H0	46
63	Dose−toxicity scenario 1	Dose−efficacy scenario 1	0.50	CA	H0	46
64	Dose−toxicity scenario 1	Dose−efficacy scenario 1	0.75	CA	H0	46
65	Dose−toxicity scenario 1	Dose−efficacy scenario 1	1.00	CA	H0	47
66	Dose−toxicity scenario 1	Dose−efficacy scenario 1	0.00	PA	H0	45
67	Dose−toxicity scenario 1	Dose−efficacy scenario 1	0.25	PA	H0	46
68	Dose−toxicity scenario 1	Dose−efficacy scenario 1	0.50	PA	H0	46
69	Dose−toxicity scenario 1	Dose−efficacy scenario 1	0.75	PA	H0	46
70	Dose−toxicity scenario 1	Dose−efficacy scenario 1	1.00	PA	H0	47
71	Dose−toxicity scenario 1	Dose−efficacy scenario 1	0.00	CD	H0	45
72	Dose−toxicity scenario 1	Dose−efficacy scenario 1	0.25	CD	H0	46
73	Dose−toxicity scenario 1	Dose−efficacy scenario 1	0.50	CD	H0	47
74	Dose−toxicity scenario 1	Dose−efficacy scenario 1	0.75	CD	H0	47
75	Dose−toxicity scenario 1	Dose−efficacy scenario 1	1.00	CD	H0	47
76	Dose−toxicity scenario 1	Dose−efficacy scenario 2	0.00	CA	H0	46
77	Dose−toxicity scenario 1	Dose−efficacy scenario 2	0.25	CA	H0	47
78	Dose−toxicity scenario 1	Dose−efficacy scenario 2	0.50	CA	H0	47
79	Dose−toxicity scenario 1	Dose−efficacy scenario 2	0.75	CA	H0	48
80	Dose−toxicity scenario 1	Dose−efficacy scenario 2	1.00	CA	H0	48
81	Dose−toxicity scenario 1	Dose−efficacy scenario 2	0.00	PA	H0	46
82	Dose−toxicity scenario 1	Dose−efficacy scenario 2	0.25	PA	H0	47
83	Dose−toxicity scenario 1	Dose−efficacy scenario 2	0.50	PA	H0	47
84	Dose−toxicity scenario 1	Dose−efficacy scenario 2	0.75	PA	H0	48
85	Dose−toxicity scenario 1	Dose−efficacy scenario 2	1.00	PA	H0	48
86	Dose−toxicity scenario 1	Dose−efficacy scenario 2	0.00	CD	H0	46
87	Dose−toxicity scenario 1	Dose−efficacy scenario 2	0.25	CD	H0	47
88	Dose−toxicity scenario 1	Dose−efficacy scenario 2	0.50	CD	H0	47
89	Dose−toxicity scenario 1	Dose−efficacy scenario 2	0.75	CD	H0	47
90	Dose−toxicity scenario 1	Dose−efficacy scenario 2	1.00	CD	H0	47
91	Dose−toxicity scenario 2	Dose−efficacy scenario 1	0.00	CA	H0	46
92	Dose−toxicity scenario 2	Dose−efficacy scenario 1	0.25	CA	H0	47
93	Dose−toxicity scenario 2	Dose−efficacy scenario 1	0.50	CA	H0	47
94	Dose−toxicity scenario 2	Dose−efficacy scenario 1	0.75	CA	H0	48
95	Dose−toxicity scenario 2	Dose−efficacy scenario 1	1.00	CA	H0	48
96	Dose−toxicity scenario 2	Dose−efficacy scenario 1	0.00	PA	H0	46
97	Dose−toxicity scenario 2	Dose−efficacy scenario 1	0.25	PA	H0	47
98	Dose−toxicity scenario 2	Dose−efficacy scenario 1	0.50	PA	H0	47
99	Dose−toxicity scenario 2	Dose−efficacy scenario 1	0.75	PA	H0	47
100	Dose−toxicity scenario 2	Dose−efficacy scenario 1	1.00	PA	H0	48
101	Dose−toxicity scenario 2	Dose−efficacy scenario 1	0.00	CD	H0	46
102	Dose−toxicity scenario 2	Dose−efficacy scenario 1	0.25	CD	H0	47
103	Dose−toxicity scenario 2	Dose−efficacy scenario 1	0.50	CD	H0	48
104	Dose−toxicity scenario 2	Dose−efficacy scenario 1	0.75	CD	H0	48
105	Dose−toxicity scenario 2	Dose−efficacy scenario 1	1.00	CD	H0	48
106	Dose−toxicity scenario 2	Dose−efficacy scenario 2	0.00	CA	H0	47
107	Dose−toxicity scenario 2	Dose−efficacy scenario 2	0.25	CA	H0	48
108	Dose−toxicity scenario 2	Dose−efficacy scenario 2	0.50	CA	H0	48
109	Dose−toxicity scenario 2	Dose−efficacy scenario 2	0.75	CA	H0	49
110	Dose−toxicity scenario 2	Dose−efficacy scenario 2	1.00	CA	H0	49
111	Dose−toxicity scenario 2	Dose−efficacy scenario 2	0.00	PA	H0	47
112	Dose−toxicity scenario 2	Dose−efficacy scenario 2	0.25	PA	H0	48
113	Dose−toxicity scenario 2	Dose−efficacy scenario 2	0.50	PA	H0	48
114	Dose−toxicity scenario 2	Dose−efficacy scenario 2	0.75	PA	H0	49
115	Dose−toxicity scenario 2	Dose−efficacy scenario 2	1.00	PA	H0	49
116	Dose−toxicity scenario 2	Dose−efficacy scenario 2	0.00	CD	H0	47
117	Dose−toxicity scenario 2	Dose−efficacy scenario 2	0.25	CD	H0	49
118	Dose−toxicity scenario 2	Dose−efficacy scenario 2	0.50	CD	H0	48
119	Dose−toxicity scenario 2	Dose−efficacy scenario 2	0.75	CD	H0	49
120	Dose−toxicity scenario 2	Dose−efficacy scenario 2	1.00	CD	H0	49
